# Acetic Acid in Erysipelas

**Published:** 1859-11

**Authors:** Wm. Hauser

**Affiliations:** Spier’s Turnout, Ga.


### ARTICLE III.

*Acetic Acid in Erysipelas.* By Wm. Hauser, M. D., of Spier's Turnout, Ga.

When I settled in this neighborhood, in 1846, a case of Erysipelas seldom, indeed never, came under my notice; but during the last six years, cases have been of more or less frequency.

I shall not burden the Journal with a disquisition on the various phases of this hydra-disease, my object being to call attention to Acetic Acid as a remedy in the fiery, violent, phlegmonous variety of it. In the 2d No. of the Oglethorpe Medical Journal, for the present year, there is an interesting article on this subject, by Dr. B. W. Hardee, of Savannah. I read that article with interest, and have since had occasion to make trial of the remedy. Indeed, for several years I have regarded Erysipelas as the result of a super-alkaline condition of the system, and have found that treatment based upon that idea, has been successful in every case but one; and in that one, gangrene of the cervical tissues had supervened ere the plan could be tried. Dr. John Bell, of Philadelphia, and Dr. H. L. Byrd, of Savannah, have written on the use of Tr. Murias Ferri in this disease; and I attribute their success to the acid character of the medicine.

But to a case treated with Acetic Acid: Mrs. S., æt 50, of plethoric habit and sanguine billious temperament, was attacked with painful, burning, rapidly spreading Erysipelas on the left ankle. I prescribed Epsom Salts with plenty of vinegar—an indefinite quantity—and applied sugar of lead water to the part. As the skin was dry and hot I gave Tr. Guaiac and Nitras Potas, till diaphoresis came on. On the second day she was finely convalescent, and rapidly recovered.

My experience with this article in Erysipelas is small, but decidedly satisfactory so far as it has gone; and I hope this article may provoke wiser and more experienced men to give us, in the Journal, the result of their experience.

